# Reduced activity of parvalbumin-positive interneurons in the posterior parietal cortex causes visually dominant multisensory decisions in freely navigating mice

**DOI:** 10.1186/s13041-022-00968-x

**Published:** 2022-10-12

**Authors:** Ilsong Choi, Seung-Hee Lee

**Affiliations:** 1 Department of Biological Sciences, KAIST, Daejeon, 34141 Republic of Korea; 2 Center for Synaptic Brain Dysfunctions, Institute for Basic Science (IBS), Daejeon, 34141 Republic of Korea

**Keywords:** Multisensory integration, Posterior parietal cortex, Parvalbumin-positive neurons, In vivo calcium imaging, Optogenetics

## Abstract

Multisensory integration is vital for animals to make optimal decisions in a complicated sensory environment. However, the neural mechanisms for flexible multisensory behaviors are not well understood. Here, we found that mice exhibit auditory-dominant decisions in the head-fixed and stationary state and switch to make visual-dominant decisions in the freely navigating state to resolve audiovisual conflicts. To understand the neural mechanism of the state-dependent switch in multisensory decisions, we performed in vivo calcium imaging of parvalbumin-expressing (PV^+^) inhibitory neurons in the posterior parietal cortex (PPC), which are known to mediate auditory dominance in the resolution of audiovisual conflicts, in mice on the treadmill. In the stationary state, the PPC PV^+^ neurons showed similar amounts of evoked activity in responses to auditory and visual stimuli and enhanced responses to the multisensory audiovisual stimuli. Conversely, when mice were running on a treadmill, the PV^+^ neurons lost auditory responses and did not show any multisensory enhancement in their activity. When we optogenetically activated the PPC PV^+^ neurons in mice freely navigating the T-maze, the mice made more auditory-dominant decisions without changes in unisensory decisions. Our data demonstrate that the PPC PV^+^ neurons lost their ability to integrate auditory information with the visual one during active navigation. This modulation of the PPC PV^+^ neuron activity is critical for animals to make adaptive multisensory decisions according to their behavioral states.

**Supplementary Information:**

The online version contains supplementary material available at 10.1186/s13041-022-00968-x.

## Main text

Animals receive sensory information from multiple sensory modalities in the natural environment. Sensory inputs are first processed in the sensory cortices, and this sensory processing is modulated according to the animal’s internal state [1]. Multisensory integration, a brain process combining sensory information across modalities, is a vital process for animals to make optimal decisions in the complicated sensory world [2–4]. Previous studies reported that animals often make a biased perception toward one modality among all types of sensory inputs, showing sensory dominance in their perception during multisensory integration [5–8]. We recently found that laboratory mice in a head-fixed state showed auditory-dominant decisions when conflicting auditory and visual stimuli were presented simultaneously. This auditory-dominant resolution of audiovisual conflicts required the activity of the parvalbumin-expressing (PV^+^) neurons in the posterior parietal cortex (PPC) [9], which is one of the key association cortical areas integrating multisensory information in mammalian brains [10–12]. Although it has been shown that unisensory processing in the sensory cortex can be modulated by locomotion [13–15], it is still unclear how locomotion affects the multisensory integration process in the association cortex [1]. In particular, different task regimes require distinct behavioral states of the animal, and thus multisensory decisions of the same animal may not be the same in different tasks.

**Fig. 1:**
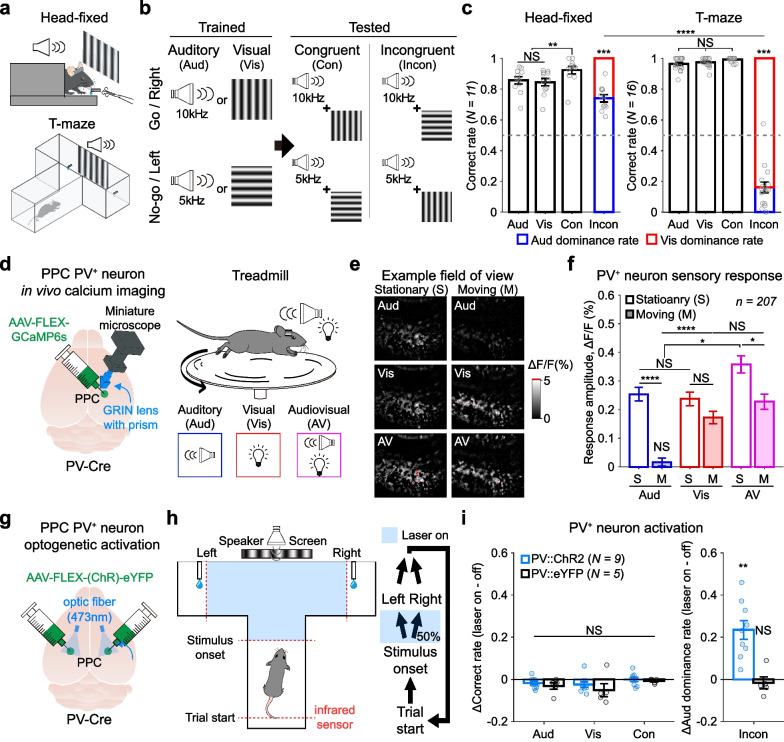
Locomotion enhances visual dominant decisions of mice under audiovisual conflicts by reducing auditory responses in PV^+^ neurons of the PPC. **a** Schematic illustration of the audiovisual discrimination task in head-fixed mice (top) and freely-navigating mice in the T-maze (bottom). **b** Auditory and visual stimuli used in training (left) and testing (right) audiovisual discrimination behaviors. **c** Behavioral results of mice under the audiovisual task in the head-fixed condition (left) and in the freely-navigating state in the T-maze (right). Colors in incongruent trials indicate auditory (blue) and visual (red) dominance rates. Circles indicate data from individual mice. Asterisks in incongruent trials indicate statistical comparisons with the chance level (0.5, gray dashed line). **d** Schematic illustration of in vivo calcium imaging from PV^+^ neurons in the PPC (left) and the stimuli presented to a mouse on the non-motorized treadmill (right). **e** Example field of views showing calcium activities of PPC PV^+^ neurons during 1 s after the stimulus onset. Red pixels indicate the fluorescent intensity became 5% brighter than the baseline. **f** Mean response amplitudes (0–1 s) of the PPC PV^+^ neurons to the auditory, visual, or audiovisual stimuli at stationary or moving conditions. **g** Schematic illustration of optogenetic activation of the PV^+^ neurons in the bilateral PPC. **h** Schematic illustration of how optogenetic stimulus is given during a trial. Blue shades indicate the area (left) and the time (right) where and when the optogenetic activation was given. **i** Changes in the correct rates (left) and auditory dominance rate (right) of mice by the optogenetic activation of the PPC PV^+^ neurons in the T-maze. Statistical comparison with chance level (‘0’) were indicated above each bar. Data are presented as means ± SEM. NS (non-significant); *P < 0.05; **P < 0.01; ***P < 0.001; ****P < 0.0001. Wilcoxon signed-rank tests, Mann–Whitney U tests for the behavior data (**c** and **i**), and Student’s t-test for the calcium imaging data (**f**) with the Bonferroni correction

To investigate the state-dependent changes in multisensory decisions, we trained mice to discriminate audiovisual stimuli under two different experimental setups—one is the head-fixation setup with a go/no-go licking detection system, and the other is the T-maze setup with a left/right choice system (Fig. 1a). In these systems, we trained a group of mice in the head-fixed condition and another group in the freely-navigating condition to discriminate the same audiovisual stimuli. Two visual and two auditory stimuli were randomly presented during the training until mice became experts in discriminating both auditory and visual stimuli. We then presented congruent (same meaning in the auditory and visual stimuli) or incongruent (opposite meaning in the auditory and visual stimuli) audiovisual stimuli (Fig. 1b). Since we used salient stimuli in both auditory and visual conditions (see Additional file 1 for the detailed methods), mice learned the task well and showed similar levels of unisensory discrimination performances in both head-fixed and freely navigating conditions (see correct rates of Aud and Vis in Fig. 1c). Interestingly, however, head-fixed mice showed auditory-dominant decisions, but navigating mice showed strong visual dominance to resolve audiovisual conflicts in the incongruent trials (Fig. 1c). These results indicate that changes in behavioral states switch the dominant modality that mice more relied on during the resolution of audiovisual conflicts.

Compared to the head-fixed state, one main change that happened during navigation was the increase in locomotion during the task. We previously found that the activation of PV^+^ neurons in the PPC is critical for auditory-dominant resolution of audiovisual conflicts [9]. We thus asked whether locomotion alters the sensory-evoked activity of PV^+^ neurons in the PPC. We measured sensory responses of the PV^+^ neurons in the PPC by in vivo calcium imaging in head-fixed and untrained mice on a non-motorized treadmill using a miniaturized one-photon fluorescence microscope (Fig. 1d) (see Additional file 1 for the detailed methods and Additional file 2 for the immunohistochemical confirmation). In the stationary state, the PPC PV^+^ neurons showed similar levels of activity in response to the visual and the auditory stimuli, and their activity was even more enhanced for the audiovisual multisensory stimuli (Fig. 1e, f). Interestingly, the auditory response of the PPC PV^+^ neurons was significantly reduced and almost disappeared when mice were running on the treadmill (Fig. 1e, f). Only the visual response remained in the PPC PV^+^ neurons during locomotion (Fig. 1f).

Finally, we tested whether activating PV^+^ neurons in freely navigating mice in the T-maze switches visual dominance to auditory dominance during the resolution of audiovisual conflicts. To activate PV^+^ neurons in the PPC bilaterally, we injected the adeno-associated virus (AAV) expressing channelrhodopsin (ChR2) in a Cre-dependent manner into the PPC of the PV-Cre mice in both hemispheres (Fig. 1g) (see Additional file 2 for the immunohistochemical confirmation). We then optogenetically activated PV^+^ neurons in task-performing mice by delivering the blue lights on the PPC randomly in 50% of the trials from the stimulus onset until mice chose the left or right arms (Fig. 1h). Activation of the PPC PV^+^ neurons did not affect the decisions of mice to unisensory (auditory or visual) or congruent audiovisual stimuli (Fig. 1i) (see Additional file 2 for the raw data of correct rate). However, it significantly increased the audition-dominant decisions of mice in incongruent trials (Fig. 1i).

In conclusion, our study demonstrates that behavioral states determine dominance between auditory and visual decision-making when these sensory modalities conflict with each other. An increase in locomotion during the task reduced the activity of PV^+^ neurons in the PPC. We previously found that the PV^+^ neurons mediated auditory-to-visual suppression in the PPC, which led to the auditory-dominant decisions in the head-fixed mice [9]. In this study, we newly found that locomotion reduced the activity of PV^+^ neurons. This would lead to an increase in visual representation in the PPC, which may lead to the visually dominant decisions in mice. Supporting this, optogenetic activation of the PV^+^ neurons in the PPC increased auditory-dominant decisions in mice navigating the T-maze (Fig. 1i and Additional file 2: Fig. S2). However, our optogenetic experiment was not sufficient to switch the visual dominance to the auditory dominance completely in navigating mice. This may be due to the limitation of the optogenetic experiment in activating the PV^+^ neurons, and thus the enhanced visual representation in the PPC may not be fully suppressed in this experimental condition. It is also possible that the locomotion may modulate other types of interneurons in the cortex, such as the vasoactive intestinal peptide-expressing (VIP^+^) or the somatostatin-expressing (SOM^+^) neurons, to induce visual dominance in mice. Our data suggest that mice rely more on visual information than auditory one in navigating conditions but switch to rely more on auditory information in stationary states to resolve the conflicts between the modalities. This adaptive multisensory processing can be explained by the locomotion-induced suppression of the activity of PV^+^ inhibitory neurons in the PPC. Overall, our study gives ethological insights into mammalian multisensory behaviors. Future studies will be required to fully understand how locomotion modulates the activity of inhibitory neurons in the PPC, not only PV^+^ neurons but VIP^+^ and SOM^+^ neurons.

## Supplementary Information

**Additional file 1.** Materials and methods.

**Additional file 2.** Additional figures. Data of immunohistochemistry results, and correct rate data of PPC PV^+^ activation from task-performing mice in the T-maze.

## Data Availability

The detailed methods are described in Additional file 1. All data and materials are available from the corresponding author upon request.

## Abbreviations

PV: Parvalbumin
VIP: Vasoactive intestinal peptide
SOM: Somatostatin
PPC: Posterior parietal cortex
Aud: Auditory
Vis: Visual
Con: Congruent
Incon: Incongruent
AAV: Adeno-associated virus
ChR2: Channelrhodopsin2
