# Supplementary material for: Reduced activity of parvalbumin-positive interneurons in the posterior parietal cortex causes visually dominant multisensory decisions in freely navigating mice
Source: Mol Brain. 2022 Oct 12;15:82. doi: 10.1186/s13041-022-00968-x (PMC9559816; doi:10.1186/s13041-022-00968-x)
Supplement: Supplementary file 1 — Additional file 1. Materials and methods. [file 13041_2022_968_MOESM1_ESM.docx]

Additional file.1

**Materials and Methods**

**Animals and surgery**

All experimental procedures were approved by the KAIST Institutional Animal Care and Use Committee (IACUC-16-084). Both male and female mice (P45-P180) were used in this study. Animals were maintained *ad libitum* under light (8 am-8 pm) and dark cycle (8 pm-8 am) conditions and housed under single animal per cage conditions from the start until the last day of the experiment. We used C57BL/6J wild-type (WT) mice, Pvalb^tm1(cre)Arbr^/J mice (PV-IRES-Cre, stock no. 008069, Jackson Laboratory).

To implant a head plate on the mouse head for head fixation, we anesthetized adult mice (P45-P80) with 1.5%–2% isoflurane and fixed their head on the stereotaxic apparatus. Body temperature was monitored and maintained at 37°C through a heating pad connected to a temperature sensor. The scalp was alternately sterilized with 70% ethanol and povidone-iodine solution three times, and a part of the scalp was removed with surgical scissors. The exposed skull and incision were sterilized with povidone-iodine solution and washed three times with sterilized 1X phosphate-buffered saline (PBS). Three miniature stainless steel screws (J.I. Morris) were implanted into the exposed skull with cyanoacrylate glue (Loctite super glue 401, Henkel). Finally, a customized stainless steel head plate for head fixation was glued and secured with dental acrylic (Ortho-Jet, Lang Dental) on the skull.

To express GCaMP6s in the parvalbumin-expressing (PV^+^) neurons in the right posterior parietal cortex (PPC), we incised the sterilized scalp along the anterior-posterior axis. After the disinfection of the exposed skull, we made a small craniotomy (diameter ~0.5 mm) above the injection site. We then injected ~0.5 μl of the adeno-associated virus (AAV) expressing GCaMP6s in a Cre-recombinase-dependent manner (AAV5-Syn-Flex-GCaMP6s-WPRE-SV40, UPENN) into the posterior PPC (bregma -2.15mm, lateral -1.6mm, depth -0.4mm: 0.25mm posterior from the center of the PPC) on the right hemisphere of PV-IRES-Cre mice to avoid too much expression of the GCaMP6s in the imaging area. For the injection, we used a thin pulled glass pipette (20~25μm of tip diameter, item no. 504949, WPI) installed in a Nanoliter 2010 injector (WPI) and loaded the virus solution at a speed of 24 nl/min. After the injection, the incised skin was sutured with a stitching fiber, and the sutured skin was sterilized with a povidone-iodine solution. 10-14 days after the injection, gradient reflective index (GRIN) lens implantation surgery was conducted [1]. In brief, mice were subcutaneously injected with carprofen (5mg/Kg) and dexamethasone (5mg/Kg) 1 hour before the surgery. After sterilization and removal of the scalp, 1mm^2^ of square-shaped craniotomy was made on the skull above the implantation area. A prism-attached GRIN lens (1050-004601 or 1050-004606, Inscopix) was inserted at 0.2mm anterior from the center of the PPC, where we made a small linear cut of the brain tissue with a surgical knife (10055-12, Fine Science Tools) attached to the stereotaxic apparatus. The GRIN lens was slowly inserted (-0.1mm/20 s, depth -0.8mm) according to the medial-lateral axis. The exposed brain tissue between the GRIN lens and skull was covered with Kwik-Sil (WPI), and the inserted GRIN lens was secured by applying Super-Bond (Super-Bond C&B Kit, Sun Medical Co.) and the cyanoacrylate glue over the layer of cured Kwik-Sil and joining skull. Mice were further implanted with the head plate. Mice were subcutaneously injected with carprofen and dexamethasone for the subsequent three days from the day of surgery. The field of view of each mouse was monitored periodically, and a baseplate (1050-004638, Inscopix) was fixed above the GRIN lens with dental acrylic if we observed clear neuronal activities. The baseplate was covered with a baseplate cover (1050-004639, Inscopix) to protect the lens from dirt.

For optogenetic activation of PV^+^ neurons in the bilateral PPC, we injected 0.5 μl of Cre-recombinase-dependent AAV expressing ChR2-eYFP (AAV2-EF1a-DIO-hChR2-EYFP, UNC Vector Core) or eYFP (AAV2-EF1a-DIO-EYFP, UNC Vector Core) into the bilateral PPC of PV-IRES-Cre mice. After the injection, mice were further implanted with the head plate. Mice had 14 days of viral expression before starting water restriction for behavioral training. When mice became experts (> 80% of correct rates for both auditory and visual tasks), we provided mice with sufficient water for 2 days and performed optic fiber implant surgery. Mice were anesthetized with 1.5%-2% isoflurane, and a 0.5mm-diameter craniotomy was made on the PPC. An optic fiber with a 200µm diameter was implanted into the bilateral PPC at ~0.3mm depth and glued with Kwik-Sil and cyanoacrylate glue. We recovered mice for three days after the surgery and restarted the water restriction and behavioral training.

**Behaviors and audiovisual discrimination tasks for mice**

Mice underwent water restriction (~1ml of water per day) throughout the behavioral experiment. The weight of mice under the water restriction was monitored throughout the experiments and maintained at more than 75% of the initial body weight before the water restriction.

*Head-fixed mice training*

For the presentation of the sensory stimuli to the head-fixed mice, a gamma-corrected LCD monitor (12cm x 9cm; minimal luminance: 0 lux; maximal luminance: 120 lux; refresh rate: 60Hz) was placed 10 cm away from the left eye of head-fixed mice at a 45° angle from the rostral-caudal axis (horizontal angular size was 62°). A speaker (#60108, Avisoft) was placed 10cm away from the left ear of the head-fixed mice. Visual stimuli were generated with a custom MATLAB code. Full-field drifting gratings (100% contrast, 2 Hz temporal resolution, 0.04 cycles/° spatial resolution, 60Hz refresh rate) in two directions (rightward for go; upward for no-go) were used for the visual discrimination task. Auditory stimuli were generated with a custom MATLAB code (sampling rate 119,000Hz, 16bit). Pure tones with different frequencies (10kHz for go, 5kHz for no-go) were used for the auditory discrimination task. Both sounds were calibrated at a 76dB sound pressure level (SPL) at the position where the left ear of the head-fixed mouse was. Mice were head-fixed and body-restrained in a cylindrical tube. A custom-made lickometer detected the licking behavior of task-performing mice. We delivered water reward and air punishment via solenoid valves (EV-2-24, Clippard). Presentation (Neurobehavioral Systems) operated and controlled all the hardware components through USB data acquisition devices (USB-201, Measurement Computing).

All head-fixed mice were trained and tested through the following four steps: 1) Reward conditioning; 2) Stimulus conditioning; 3) Audiovisual discrimination training; 4) Audiovisual integration test.

1) Reward conditioning: water-restricted mice could get 0.6μl of water reward whenever they licked the water port. If mice did not show any spontaneous licking behavior within the first 3 minutes after the beginning of the session, we manually delivered water through the water port until mice licked the water port. If mice got more than 0.6ml of total water reward (> 1000 licks) within the first 20 minutes without manual water delivery, we started the second training step in the next session.

2) Stimulus conditioning: auditory go stimulus, visual go stimulus, and blank stimulation were pseudo-randomly presented in the proportion of 1:1:2. If mice licked the water port during 2 s of response window period after the go stimulus offset (hit), 1μl of water reward and 2 s of reward time were presented after the first licking during response window. If mice did not lick the water port until the offset of the response window (miss), 1μl of water reward was delivered at the end of the response window. If mice licked the water port for the no stimulation trial (false-alarm, FA), 6 s of additional time-out period was given. Before starting the next trial, there were 4 s of extra time delay for the inter-trial interval. Task sessions automatically ended when mice consecutively missed 30 auditory or visual go stimuli. When mice showed more than 80% of hit rates for both visual and auditory go stimuli, we started the third training step in the next session.

3) Audiovisual discrimination training: 4 stimuli (auditory go, auditory no-go, visual go, visual no-go) were pseudo-randomly presented in equal proportion. There was no water reward in miss trials, and 400ms of air puff punishment was delivered to the right cheek of mice with the time-out period (6 s) in FA trials. There was no water reward in correct rejection trials (CR) which mice did not show licking behavior after the no-go stimulus presentation. Task sessions automatically ended when mice consecutively missed 10 auditory or visual go stimuli. If mice achieved correct rates higher than 80% for both auditory and visual discrimination, we considered mice learned the discrimination tasks.

4) Audiovisual integration test: auditory stimuli, visual stimuli, congruent stimuli, and incongruent stimuli were pseudo-randomly presented in the proportion of 3:3:1:1. There was no water reward and air puff punishment for the licking in the incongruent trials. Each session was finished when mice showed three consecutive miss trials or elapsed session time became 1 hour.

We calculated hit rate, auditory dominance rate, and correct rate as follows:

*Hit rate = #Hit trials / #Go trials*

*Correct rate = (#Hit trials + #Correct rejection trials) / #Total trials*

*Auditory dominance rate = #Auditory following trials / #Incongruent trials*

We excluded data from the test sessions, in which mice discriminated one-modality stimuli better than the other-modality ones, i.e., sessions with |*auditory correct rate – visual correct rate*| > 5%.

*Freely navigating mice training in the T-maze setup*

For the T-maze experiment setup, a gamma-corrected LCD monitor (32cm x 24cm; minimal luminance: 0 lux; maximal luminance: 120 lux; refresh rate: 60Hz) was placed 30 cm away from the stimulus onset zone and 23.5 cm away from the decision point in the T-maze. The horizontal angular size was from 56° at the stimulus onset zone to 68.4° at the decision zone. The mean horizontal angular size was 62.2°, which was close to 62° from the head-fixed setup. The same full-field drifting gratings used in the head-fixed setup were used for the visual discrimination task (rightward stimuli for right; upward stimuli for left). The speaker (#60108, Avisoft) was placed above the visual screen. For the auditory discrimination task, the pure tones used in the head-fixed setup were used (10kHz for right, 5kHz for left). Both sounds were calibrated from 74dB SPL at the stimulus onset to 78dB SPL at the decision zone. A custom-made circuit detected the navigating path of task-performing mice in the T-maze with 4 sets of infrared sensors and emitters: one at the end of the corridor for the trial start, one at the middle of the corridor for the stimulus onset, and each at the end of the left or the right arm of the T for measuring choice of mice. We delivered a water reward at the end of the left and right arms after the sensors and emitters via solenoid valves (EV-2-24, Clippard). All the hardware components were operated and controlled by a customized LabView scenario (National Instruments Corporation) with USB data acquisition devices (PCA-6601, National Instruments Corporation).

All mice were trained and tested through the following four steps: 1) Reward conditioning; 2) Stimulus conditioning; 3) Audiovisual discrimination training; 4) Audiovisual integration test.

1) Reward conditioning: water-restricted mice could get 4μl of water reward whenever they made a routine which visiting one of the water ports after entering the trial start zone. If mice showed more than 2 routines per min, mice were moved on to the second step in the next session.

2) Stimulus conditioning: the auditory and visual stimuli were pseudo-randomly presented with equal ratios when mice passed the stimulus onset zone after visiting the trial start zone. 4μl of water reward was simultaneously presented with the stimulus at the water port to which mice should go. Each trial ended when mice chose one of the water ports after the stimulus onset. Mice were trained with the stimulation condition setup for 3 sessions and moved on to the third step in the next session.

3) Audiovisual discrimination training: the task scheme was the same as the stimulus conditioning, but a water reward was delivered only when mice made the correct decision. If mice achieved correct rates higher than 80% for both auditory and visual discrimination, we considered mice learned the discrimination tasks.

4) Audiovisual integration test: auditory stimuli, visual stimuli, congruent stimuli, and incongruent stimuli were pseudo-randomly presented in the proportion of 1:1:1:1. There was no water reward in the incongruent trials. Each session was finished when mice were trained with 192 trials or elapsed session time became 1 hour. We excluded data from the test sessions in which mice performed better in discriminating one modality than the other, i.e., sessions with |*auditory correct rate – visual correct rate*| > 5%.

For the optogenetic activation experiment of PV^+^ neurons in the bilateral PPC, we delivered 1.5 s of a continuous blue laser (1~1.5mW, 473nm, Shanghai Laser & Optics Century) from the stimulus onset zone in randomly chosen 50% of the trials. The mean elapsed time from the stimulus zone to the water port was less than 1 s.

***In vivo* calcium imaging experiments**

We performed *in vivo* calcium imaging experiment using a miniature fluorescence microscope (Inscopix) in the head-fixed and untrained mice on the custom-made treadmill system. An optical mouse (G100s, Logitech) was placed beside a spinning disk of the treadmill to measure the locomotion of mice across imaging sessions. The disk rotation during locomotion was converted to digitalized signals by the optical mouse. Real-time locomotion speed was calculated based on the digitalized signal from the optical mouse by a customized LabView code (sampling rate: 40Hz) through a USB data acquisition device (USB-6001, National Instruments). GCaMP calcium images were acquired in the PPC at 20 frames per second using nVista HD software (Inscopix). In addition, we cropped unnecessary imaging areas which had no neuronal signal. Different LED powers (0.2 ~ 0.5mW) and gain values (1 ~ 4) were used according to the overall brightness of GCaMP signals.

To prevent the bleaching of GCaMP signal during extended imaging sessions, we conducted imaging experiments of stationary sessions and moving sessions for two consecutive days in random order. To measure sensory responses of neurons from the untrained mice, the mice were head-fixed on the treadmill 10 minutes before the start of the imaging experiment. We presented 5kHz pure-tone (76dB SPL, 0.5 s duration), full-field flash (120lux, 0.5 s duration), and audiovisual stimulus (5kHz pure-tone + full-field flash) pseudo-randomly in equal proportion. We presented stimuli when mice were stationary (average speed < 1cm/s for 1 s) in the stationary sessions. In the moving sessions, we presented stimuli when mice ran faster than the threshold (average speed > 3cm/s for 1 s). The minimal inter-stimulus interval time was 8 s, and the duration of the interval was dependent on the locomotion state of the mice.

**Imaging data analysis**

The acquired image data were processed using Inscopix Data Processing Software (IDPS, Inscopix). Images were spatially downsampled by a factor of 4 and spatially filtered (highCutoff: 0.5; lowCutoff: 0.005). Filtered images were corrected for lateral motion (IDPS implementation). Motion-corrected images were converted to ΔF/F images where F is the average intensity of each pixel throughout the whole imaging time. The boundary of a cell was identified by principal component analysis - independent component analysis (PCA-ICA) over ΔF/F images (IDPS implementation). We then extracted calcium signals by applying manually inspected cell boundaries from PCA-ICA to the motion-corrected images and exporting average intensities within cell boundaries. The identity of neurons across two imaging sessions was identified using longitudinal registration (IDPS implementation), and only neurons found in both imaging sessions were used for further analysis. The exported calcium data were analyzed using custom MATLAB code. Extracted calcium response data were converted to ΔF/F for each neuron. ΔF/F was calculated as $\Delta F/F = (F - F0)/F0$, where F0 is the average fluorescence of a neuron across the whole imaging time. All the analyses were conducted with ΔF/F value.

To exclude possible contamination of sensory responses by locomotion, only trials without locomotion onset after the stimulus onset (*average locomotion speed during 0 ~ 0.5 s < 0.5 cm/s and average locomotion speed during 0.5 ~ 3 s < 3 cm/s*) were further analyzed in the stationary sessions. Response amplitude of each trial was calculated by subtracting average baseline activity (-0.2 ~ 0 s from the stimulus onset) from average activity during the first 1 s after the stimulus onset. Trial-averaged response amplitude was used as the response amplitude of each neuron.

**Histology and Immunohistochemistry**

We performed histology experiments to confirm imaging sites after in vivo calcium imaging. Mice were anesthetized with avertin (2,2,2-Tribromoethanol (Sigma-Aldrich), 125-250mg/kg, intraperitoneally) and transcardially perfused with 15ml of PBS followed by 15ml of 4% paraformaldehyde (PFA, w/v in PBS). Brain samples were post-fixed for 4 hours in the PFA at 4℃, then washed 3 times for 10 minutes with PBS. After washing, the brain samples were put in 30% sucrose solution (w/v in PBS) for 2-4 days. When the brain samples sank, they were embedded within an optimal cutting temperature medium (Tissue-Tek O.C.T. Compound, Sakura Finetek) and rapidly frozen at -80℃. The frozen brain samples were sectioned into 40μm (for immunostaining, 20μm) thickness in a coronal direction using a cryocut (Leica). We washed brain slices 3 times for 10 minutes with PBS and mounted them with an anti-bleaching mounting medium with 4′,6-diamidino-2-phenylindole (DAPI) (Vector Labs). For the immunostaining of PV, we permeabilized the brain sections for 30min with 0.3% Triton X-100 (w/v in PBS) at room temperature. After following washes of 3 times for 10 minutes with PBS, we incubated the slices in the blocking solution (2% w/v normal donkey serum (NDS) in PBS) for 2 hours at room temperature. The samples were incubated 48 hours at 4℃ with mouse anti-PV antibody (PV27, swant, 1:500 dilution in the blocking solution). After the treatment of the primary antibody, we washed the samples 3 times for 10 minutes with PBS. We then treated Alexa Fluor 594 donkey anti-rabbit IgG (A21207, Invitrogen, 1:500 dilution in PBS) for 2 hours at room temperature. After that, we washed the samples with PBS 3 times for 10 minutes and mounted them with anti-bleaching media with DAPI staining. Fluorescent images of brain slices were taken by slide scanner (Zeiss Axio Scan. Z1) or confocal microscope (Nikon A1 HD25).

**Statistical analysis**

We used custom-written codes in MATLAB (Mathworks) for data analysis. All data were displayed as mean ± standard error of the mean (SEM). ‘N’ indicates the number of mice or sessions, and ‘n’ indicates the number of neurons. Statistical tests of behavior data were evaluated through Wilcoxon signed-rank test (paired data or comparing with baseline) or Mann-Whitney U-test (unpaired data) with the Bonferroni correction. Statistical tests of calcium imaging data were evaluated through Student’s t-test with the Bonferroni correction. All the statistical significances of the datasets are shown as NS (not significant), *, **, ***, and **** indicate *P*≥ 0.05, *P< 0.05, P*< 0.01, *P*<0.001, and *P*<0.0001.

**Reference**

1. Gulati S, Cao VY, Otte S: Multi-layer Cortical Ca2+ Imaging in Freely Moving Mice with Prism Probes and Miniaturized Fluorescence Microscopy. *Jove-J Vis Exp* 2017(124).
