# Supplementary material for: Reduced activity of parvalbumin-positive interneurons in the posterior parietal cortex causes visually dominant multisensory decisions in freely navigating mice
Source: Mol Brain. 2022 Oct 12;15:82. doi: 10.1186/s13041-022-00968-x (PMC9559816; doi:10.1186/s13041-022-00968-x)
Supplement: Supplementary file 2 — Additional file 2. Additional figures. Data of immunohistochemistry results, and correct rate data of PPC PV+ activation from task-performing mice in the T-maze. [file 13041_2022_968_MOESM2_ESM.docx]

Additional file 2


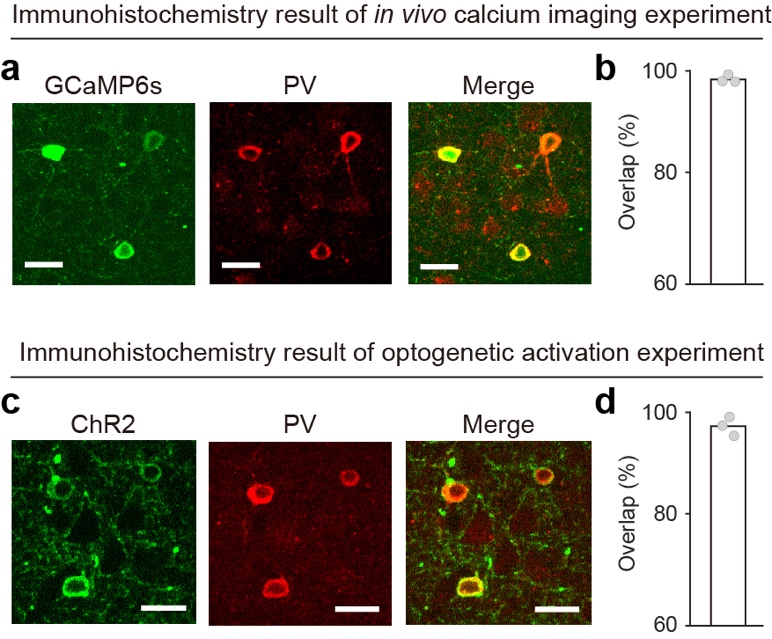


**Figure S1.** Immunohistochemistry of the PPC PV^+^ neurons expressing GCaMP6s and ChR2. **a** GCaMP6s (green) expressing PV^+^ neurons were immunostained for PV (red) in a brain slice of the PPC. Scale bars, 50 μm. **b** Percentage of GCaMP6s-expressing neurons that were immunostained for PV. Circles indicate individual mouse data. **c** ChR2 (green) expressing PV^+^ neurons immunostained for PV (red) in a brain slice of the PPC. Scale bars, 50 μm. **d** Percentage of ChR2-expressing neurons that were immunostained for PV. Circles indicate individual mouse data.

**
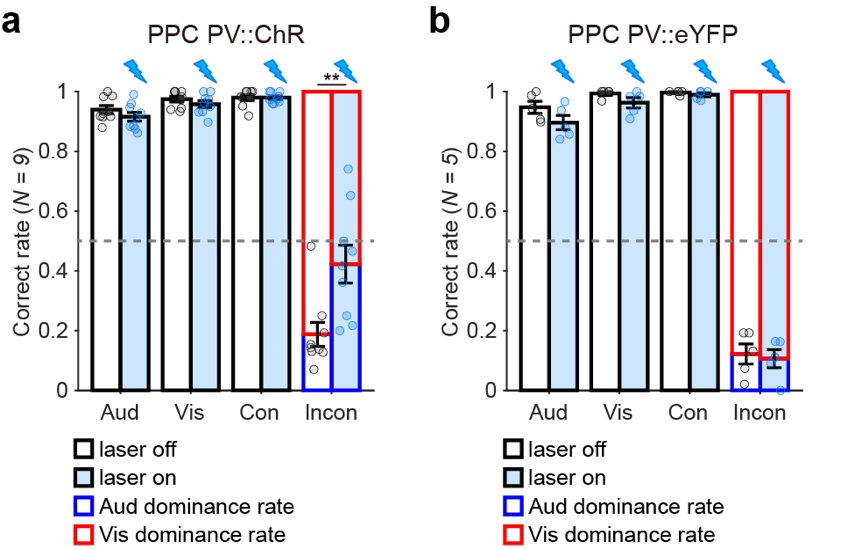
**

**Figure S2.** Changes of audiovisual discrimination performance by optogenetic activation of the PPC PV^+^ neurons in mice freely navigating the T-maze. **a** Correct rates, auditory dominance rates (blue edge), and visual dominance rates (red edge) of laser-off trials (empty bars) and laser-on trials (bars filled with light blue) from PV::ChR mice (N = 9). Circles, individual mice. **b** Same as **a**, but for PV::eYFP mice (N = 5). Data are presented as means ± SEM. **P < 0.01. Wilcoxon signed-rank test with the Bonferroni correction.
